# Electroacupuncture on GB acupoints improves osteoporosis via the estradiol–PI3K–Akt signaling pathway

**DOI:** 10.1515/biol-2022-0978

**Published:** 2024-11-19

**Authors:** Xinyu Wang, Xiyu Zeng, Yu Long, Yanfei Du, Chang Li, Hua Jiang, Guang Li

**Affiliations:** Department of Cardiology, The Affiliated Hospital of Southwest Medical University and Key Laboratory of Medical Electrophysiology, Ministry of Education & Medical Electrophysiological Key Laboratory of Sichuan Province, Institute of Cardiovascular Research, Southwest Medical University, Luzhou, 646000, Sichuan, China; Department of Acupuncture, School of Traditional Chinese Medicine Combined with Western Medicine, Southwest Medical University, Luzhou, 646000, Sichuan, China

**Keywords:** osteoporosis, electroacupuncture, PI3K-Akt, oxidative stress, estradiol

## Abstract

Recent studies have reported that electroacupuncture (EA) can treat osteoporosis, but most of which were based on the “kidney governing bones” theory. However, the ancient Chinese medical textbook *Huangdi Neijing* pointed out that “Gallbladder Meridian of Foot Shaoyang” correlates with bone diseases, including osteoporosis, although the therapeutic regimens were lost after the Tang Dynasty. Here, we explored whether EA at GB points improves osteoporosis and its underlying mechanism. We constructed ovariectomized mice and treated them with EA at GB30 (*Huantiao*), GB34 (*Yanglingquan*), and GB39 (*Xuanzhong*) acupoints. EA treatment significantly improved bone parameters in osteoporotic mice, as evidenced by micro-computed tomography and histological assessment. Additionally, EA treatment elevated the serum levels of estradiol and SOD that were downregulated in osteoporotic mice. Transcriptome and qPCR results verified that EA treatment upregulated the expression of genes associated with bone formation. Moreover, transcriptome analysis revealed differential enrichment of the PI3K–Akt pathway. Furthermore, Western blot analysis demonstrated that estradiol partially counteracted a reduction in p-AKT expression induced by hydrogen peroxide. These findings indicate that EA treatment increases serum estradiol levels in mice, thus inhibiting osteoporosis induced by oxidative stress. This effect is achieved by activating the PI3K–Akt signaling pathway.

## 1 Introduction

Osteoporosis is a skeletal disorder characterized by compromised bone strength, which increases an individual’s susceptibility to fractures [1]. Fractures considerably impact mortality, morbidity, and medical expenses globally, and their detrimental effects on public health are expected to increase in the future [2]. Both men and women experience a decline in bone mineral density (BMD) with age, beginning in midlife. Women experience a rapid phase of bone loss 5–10 years after menopause due to a sudden decrease in estrogen levels and an increase in bone turnover [3]. In men, rapid bone loss is not present due to the continuous and slow decrease of sex hormones, producing a bone loss-age curve as a gentle straight line [4]. Comparing the bone mass loss characteristics of men and women, it has been suggested that estrogen deficiency is closely related to high bone turnover metabolism.

A recent study reported that the prevalence of osteoporosis among individuals aged 40 and above in mainland China is 5% for males and 20.6% for females [5]. This poses a substantial health challenge for postmenopausal women, affecting up to 50% of this population [6]. This condition not only induces pain and inconvenience but can also result in severe disability, significantly diminishing the quality of life of patients. The pathogenesis of postmenopausal osteoporosis is intricately linked to a pronounced decrease in estrogen levels [7]. Estrogen deficiency enhances osteoclast differentiation and activity, accelerating bone resorption that outpaces bone formation. This disrupted bone metabolism is particularly evident during early menopause, leading to a reduced BMD and compromised bone microarchitecture, ultimately weakening bone strength and elevating the risk of fragility fractures [8]. To elucidate this complex pathology, ovariectomized mouse models have emerged as invaluable research tools, effectively simulating postmenopausal osteoporosis in humans. Surgical removal of the ovaries in mice rapidly decreases estrogen levels, mirroring the physiological changes experienced by postmenopausal women [9,10]. This model replicates the estrogen deficiency-induced imbalance in bone metabolism in humans, facilitating the study of osteoporosis progression and the evaluation of therapeutic interventions in a controlled setting. Consequently, ovariectomized mouse models are crucial in advancing our understanding of the underlying mechanisms of osteoporosis and for developing novel treatment strategies.

Many factors, including nutrition, cytokines, hormones, and aging, are associated with osteoporosis development [8,11,12]. Additionally, the basic loss of bone mass and reduced protein intake are critical factors contributing to the development of osteoporosis [11]. As people age, bone resorption outpaces bone formation, leading to a decrease in bone density [13]. Insufficient protein intake can further impair bone health, as proteins are essential for maintaining bone structure and function [14]. Oxidative stress is also believed to contribute to the pathogenesis of osteoporosis. Several *in vitro* and animal studies reported that oxidative stress decreased the level of bone formation by modulating the differentiation and survival of osteoblasts [15]. Moreover, osteoclasts activated by oxidative stress were shown to enhance bone resorption [16]. The balance between osteoblast formation and osteoclast resorption is related to bone development. Many studies indicated that oxidative stress plays a pivotal role in the development and progression of various types of osteoporosis [17,18]. The level of oxidative stress increases as osteoporosis advances, indicating that it plays a critical role in bone loss progression [19]. Recent studies showed that serum hydrogen peroxide (H_2_O_2_) levels are associated with decreased bone density in postmenopausal women [20]. Oxidative stress is a major pathological determinant of postmenopausal bone loss, and age-related changes, such as the loss of estrogen, exacerbate bone loss [21,22,23].

Current treatments for osteoporosis include basic treatments (e.g., calcium and vitamin D supplementation), lifestyle changes (limiting tobacco and alcohol intake, performing weight-bearing exercise, and avoiding falls or injury), and drugs (such as estrogen, parathyroid hormone, raloxifene, and denosumab). The adherence to vitamin D, calcium, and osteoporosis drugs is currently low in the investigated population [24]. Moreover, the treatment with these drugs can cause many unavoidable side effects, such as an increased risk of cardiovascular disease, gastrointestinal reactions, and rhinitis [25]. Therefore, there is an urgent need for an alternative treatment to increase bone quality and prevent fractures with a high degree of safety. In recent years, acupuncture has increasingly been used to treat osteoporosis because of its efficacy, relatively low cost, and low risk of side effects [26]. Acupuncture was widely used for treatment, along with herbal medicine, more than 2,000 years ago, and it is the second most popular supplement and alternative treatment for osteoporosis in Australia, after multivitamins [27]. Its potential benefits include increased bone density and analgesia. Clinical studies have shown that acupuncture is superior to calcium D in enhancing BMD in postmenopausal individuals [28]. Previous studies also reported that acupuncture significantly increased BMD in the lumbar vertebrae, trabecular area, and trabecular bone number while reducing the trabecular separation in postmenopausal rats [29]. Furthermore, acupuncture has been found to significantly improve osteoporosis in patients, with an increase in serum estradiol levels observed after treatment [30]. Electroacupuncture (EA), a modern technique that combines traditional acupuncture with electrotherapy, has been suggested to have beneficial effects on osteoporosis patients, based on animal experiments showing increased BMD in rats after 12 and 24 weeks [31]. The therapeutic effects of EA in the treatment of diseases such as ischemic stroke, cerebral hemorrhage, and spinal cord injury are achieved by inhibiting oxidative stress [32,33,34]. It is hypothesized that acupuncture therapy may have a positive impact on osteoporosis by mitigating oxidative stress; however, the precise underlying molecular mechanisms have yet to be fully understood. In this study, the therapeutic efficacy of EA on osteoporosis was assessed in ovariectomized mice, and an in-depth exploration of the underlying molecular mechanisms was conducted.

The traditional Chinese medicine (TCM) theory of “kidney controlling bones” has long been recognized as an important theoretical and clinical concept in the prevention and treatment of osteoporosis, as osteoporosis is considered an aging and degenerative disease caused by insufficient kidney qi, as described in the *Huangdi Neijing* [35]. However, this concept primarily emphasizes the relationship between organs and bones and does not directly reflect the connection between meridians and bones. The ancient Chinese books documented the “Gallbladder Meridian of Foot Shaoyang” for treating bone diseases, which specifically focuses on the relationship between meridians and bones. Unfortunately, this record was lost for thousands of years, rendering it unknown until recently [36]. In this study, we used this ancient description as the basis for selecting GB acupoints for EA treatment to explore the mechanism by which EA treats osteoporosis. This study provides a new approach to applying EA to the treatment of osteoporosis while elucidating the specific underlying molecular mechanisms and provides data supporting the clinical application of EA as a complementary therapy.

## 2 Materials and methods

### 2.1 Modeling and interventions

Forty female C57 mice (8 weeks old, weighing 20 ± 1 g) were purchased from Chengdu Dashuo Bioscience Inc. Animal feeding conditions were as follows: temperature, 20–26°C; relative humidity, 40–70%; light and dark cycle, 12 h/12 h; ordinary feed feeding; and free intake of water. Mice were randomly divided into four groups (*n* = 10 each): ovariectomized model with EA (OVX + EA), sham surgery model with EA (SHAM + EA), sham surgery model with non-acupoints (SHAM + non), and ovariectomized model with non-acupoints (OVX + non). After 1 week of adaptive training, mice were anesthetized with isoflurane. Ovariectomy was performed by ligating and excising the ovaries by laparotomy. In the sham groups, a small fat mass close to the ovaries was dissected. For the intervention, needles (Hanyi brand sterile acupuncture needles, half inch 0.18 × 13 mm, produced by Changchun Aikang Medical Equipment Inc., China) were inserted at GB30, GB34, and GB39 to a depth of 2–5 mm from the body surface. The EA instrument (New Huatuo brand electronic needle therapy instrument SDZ-II produced by Suzhou Medical Supplies Factory Inc., China) was connected to the three acupoints, a density wave was selected, the frequency was 15 Hz, the voltage was 1.5 V, and the needle was retained for 10 min. This was performed once a day, and the contralateral acupoints were taken the next day. Each treatment cycle comprised continuous intervention for 5 days, followed by rest for 2 days. There were four cycles of intervention over a total of 26 days. In the control group, three nonacupoints were targeted, and the mice underwent needling at these non-acupuncture points accompanied by electrical stimulation.


**Ethical approval:** The research related to animal use has been complied with all the relevant national regulations and institutional policies for the care and use of animals and has been approved by the Ethics Committee of Southwest Medical University (permission number: 2020323), and animals were cared according to the National Institutes of Health guide for the care and use of Laboratory animals guidelines (NIH Publications No. 8023).

### 2.2 Specimen collection, serum enzyme-linked immunosorbent assay (ELISA), and femoral morphology

At the end of 26 days, all mice were anesthetized with isoflurane. The abdomen was quickly opened, and the inferior vena cava was separated. Then, 0.5–0.9 mL of blood was collected with a disposable blood collection needle and placed in a centrifuge tube. After standing for 30 min, the blood was centrifuged at 4°C and 3,000×*g* for 15 min. The supernatant was rapidly frozen in liquid nitrogen and stored in a −80°C refrigerator for ELISA analysis (Elabscience Inc.).

For the ELISA analysis, mouse E2 antigen was coated on an enzyme-labeled plate, and anti-estradiol monoclonal antibody labeled with horseradish peroxidase (HRP) was added along with the sample or standard. The estradiol present in the sample or standard competed with the antigen estradiol for the binding site on the HRP-labeled anti-estradiol monoclonal antibody, forming a complex with the antigen estradiol-HRP on the plate. Any unbound components were washed away. A color-developing substrate (tetramethylbenzidine) was then added, which turned blue under catalysis by HRP and then became yellow upon the addition of a stop solution. Optical density values were measured at a wavelength of 450 nm using a spectrophotometer. The estradiol concentration was inversely proportional to the OD450 values. The estradiol concentration in the sample was determined by plotting a standard curve [37]. The mice were then killed by cervical dislocation, the bilateral leg bones were collected, and fur and muscle were removed from the leg bones. One intact tibia was fixed in 75% ethanol for micro-computed tomography (CT) bone density determination. After the CT scan, the tibia was used for Hematoxylin-Eosin staining and Masson staining, while the other tibia was cut frozen in liquid nitrogen and stored in a −80°C freezer for subsequent Western blot experiments.

### 2.3 RNA sequencing

MC3T3-E1 cells (ATCC, CRL-593) were cultured in α-minimum essential medium (Gibco Life Technologies Inc., USA) supplemented with 10% fetal bovine serum (HyClone, Inc., USA) and 1% penicillin and streptomycin antibiotics (Biyuntian Inc., China). After cells reached 80–90% confluence in a T-25-cm^2^ culture bottle, the cells were digested and seeded in a six-well plate. After 24 h of cell attachment, the old medium was removed, and a new medium with 100 μm H_2_O_2_ (Biyuntian Inc., China) or 10^−6^ mol/L estradiol (Sigma-Aldrich Inc., USA) was added to the treatment group, respectively, and phosphate-buffered solution (PBS) was added to the control group. After 12 h of treatment, adherent cells were washed quickly with PBS buffer, and 1 mL of TRIzol reagent (Thermo Scientific Inc., USA) was added to each well of the six-well plate and blown repeatedly with a pipette. The cells were transferred to a 1.5-mL RNase-free centrifuge tube for RNA sequencing (Novogene Inc., China). Liquid nitrogen was quick-frozen and stored in a −80℃ refrigerator for dry ice transportation.

### 2.4 Quantitative real time-PCR

We measured the expression of osteoblast formation markers by quantitative real time-PCR (qPCR). Total RNA was extracted from cells using NucleoZOL reagent (Macherey-Nagel Inc., Germany) according to the instructions. cDNA was synthesized using a high-capacity cDNA reverse transcription kit, and qPCR analysis was performed using ChamQ Universal SYBR qPCR Master Mix (Vazyme Inc., China) according to the manufacturer’s instructions. Target genes included *alpl* (F: ccaactcttttgtgccagaga, R: ggctacattggtgttgagctttt), *ibsp* (F: atggagacggcgatagttcc, R: ctagctgttacacccgagagt), *col1a1* (F: gctcctcttaggggccact, R: attggggacccttaggccat), and *runx2* (F: gactgtggttaccgtcatggc, R: acttggtttttcataacagcgga). *GAPDH* (F: tgcccccatgtttgtgatg, R: tgtggtcatgagcccttcc) served as an internal control. The relative levels of gene expression were estimated using the 2^−ΔΔCt^ method.

### 2.5 Western blotting

Cells and bone tissue were lysed with RIPA lysis buffer (Solarbio Inc.) containing a 1× protease inhibitor and phosphatase inhibitor (Thermo Scientific Inc., USA). Equivalent amounts of protein were separated by electrophoresis and transferred to a polyvinylidene fluoride membrane. The membranes were probed with anti-Akt (1:1,000; #60203-2-lg; Proteintech Inc., China) and anti-phosphorylated Akt (1:1,000; Ser473; Cell Signaling Technology Inc., USA) antibodies, followed by HRP-conjugated anti-lgG [38]. Bound antibodies were visualized using a chemiluminescent substrate (Merck Millipore, Watford, UK) and images were acquired with Quantity One software (Bio-Rad, CA, USA).

### 2.6 Statistical analysis

All experiments were performed at least three times independently. Statistical analysis was carried out using GraphPad Prism 8.0 software by one-way analysis of variance and Tukey’s multiple comparison test. Statistical significance was defined as *p* < 0.05.

## 3 Results

### 3.1 EA improves bone density

**Figure 1:**
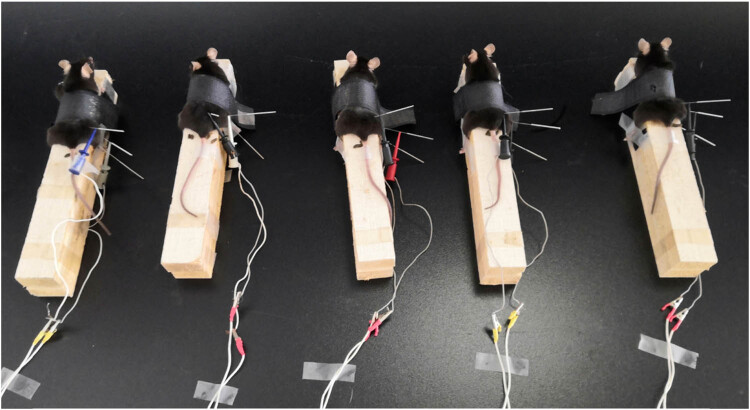
Mice in electroacupuncture (A).

**Figure 2:**
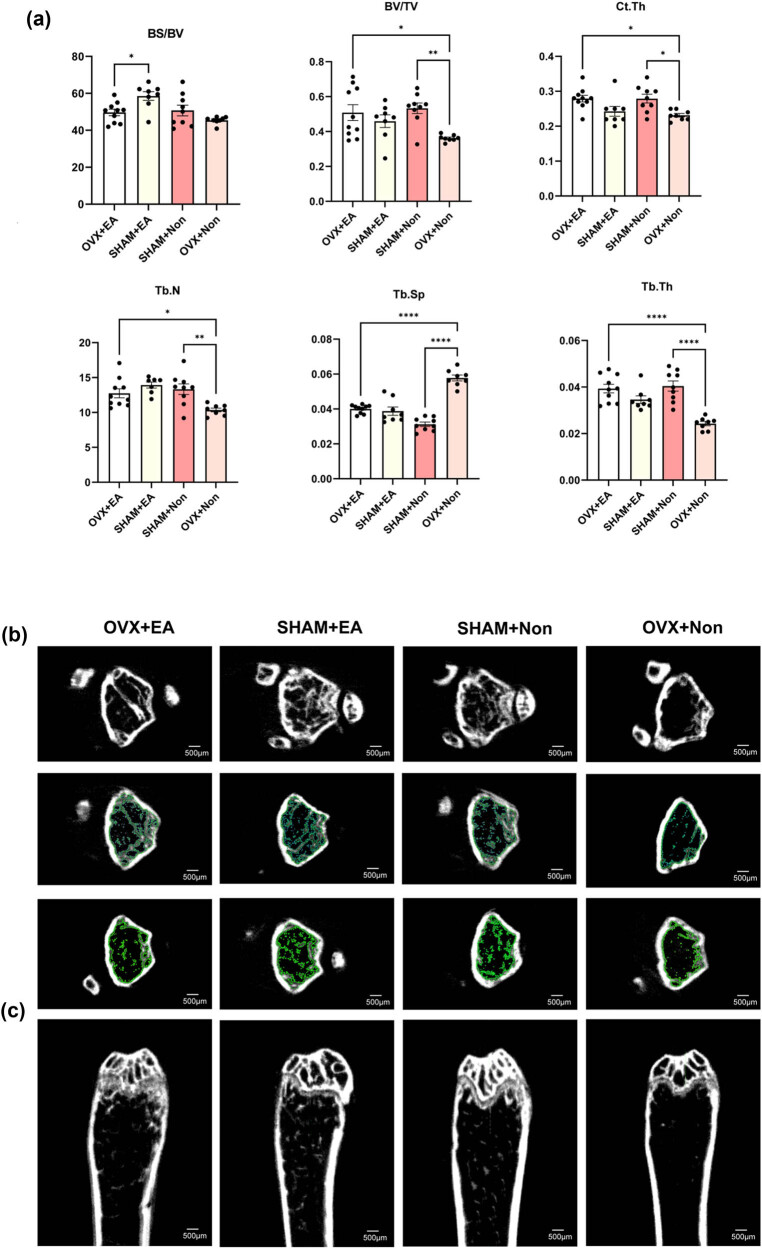
Micro-CT showed changes in bone density-related indexes in mice after EA treatment. BS/BV, bone surface area-to-bone volume ratio; BV/TV, bone volume fraction; Ct.Th, cortical bone thickness; Tb.N, number of trabecular; Tb.Sp, trabecular separation; Tb.Th, trabecular thickness. Quantitative parameters of trabecular bone and cortical bone were analyzed (a). Data are performed as mean ± SEM. *, *p* < 0.05; **, *p* < 0.01; ***, *p* < 0.001; ****, *p* < 0.0001. Transverse plane morphology of the femur micro-CT; green represents bone trabeculae from different layers (b). Sagittal plane morphology of the femur (c).

The femurs of mice were scanned using a Siemens Inveon MM scanner, which has a spatial resolution of 1.4 mm with an X-ray energy of 80 kV and 500 μA. Micro-CT is a superior technique to dual-energy X-ray absorptiometry, as it provides a more accurate reflection of changes in bone mass, BMD, and quantitative measurements of bone trabecular microstructure, including bone volume, cortical thickness, and bone strength. Therefore, micro-CT is an important tool in the study of osteoporosis, osteoarthritis, osteosclerosis, and other bone metabolic diseases for elucidating bone microstructure in animal experiments and *in vitro* human specimens [39]. After interventions in ovariectomized mice (Figure 1), indicators including bone volume fraction (BV/TV), cortical bone thickness (Ct.Th), number of bone trabecular (Tb.N), and trabecular thickness (Tb.Th) were significantly increased, while the trabecular separation degree (Tb.Sp) showed an opposite trend (Figure 2a). The same results were observed in sham mice compared to ovariectomized mice, suggesting that the osteoporosis model in our study was successful. As shown in Figure 2, the bone structure arrangement of ovariectomized mice without interventions was sparse, disordered, and discontinuous; some regions of trabeculae were disrupted or even disappeared, and the bone marrow cavity was empty (Figure 2b and c). The bone surface area-to-bone volume (BS/BV) ratio can indirectly reflect bone mass. BV/TV, which is a common index for evaluating the bone mass of cortical bone and cancellous bone. In intramedullary cancellous bone, this ratio can reflect the amount of trabecular bone mass in different samples [40]. An increase in this value indicates that bone anabolism is greater than catabolism, causing the bone mass to increase, and indirectly reflects bone metabolism. This also applies to the evaluation of bone mass and bone metabolism of cortical bone in the middle part of the long bone. Tb.N, Tb.Th, and Tb.Sp are the main indexes characterizing trabecular spatial morphology. When bone catabolism is greater than bone anabolism, such as when osteoporosis occurs, Tb.N and Tb.Th decrease, and Tb.Sp increases. These indices indicate that bone anabolism is greater than catabolism, and bone mass increases, indirectly reflecting bone metabolism. EA treatment also showed a positive effect on the number of trabeculae and their arrangement, which was further confirmed by Hematoxylin-Eosin staining and Masson staining.

**Figure 3:**
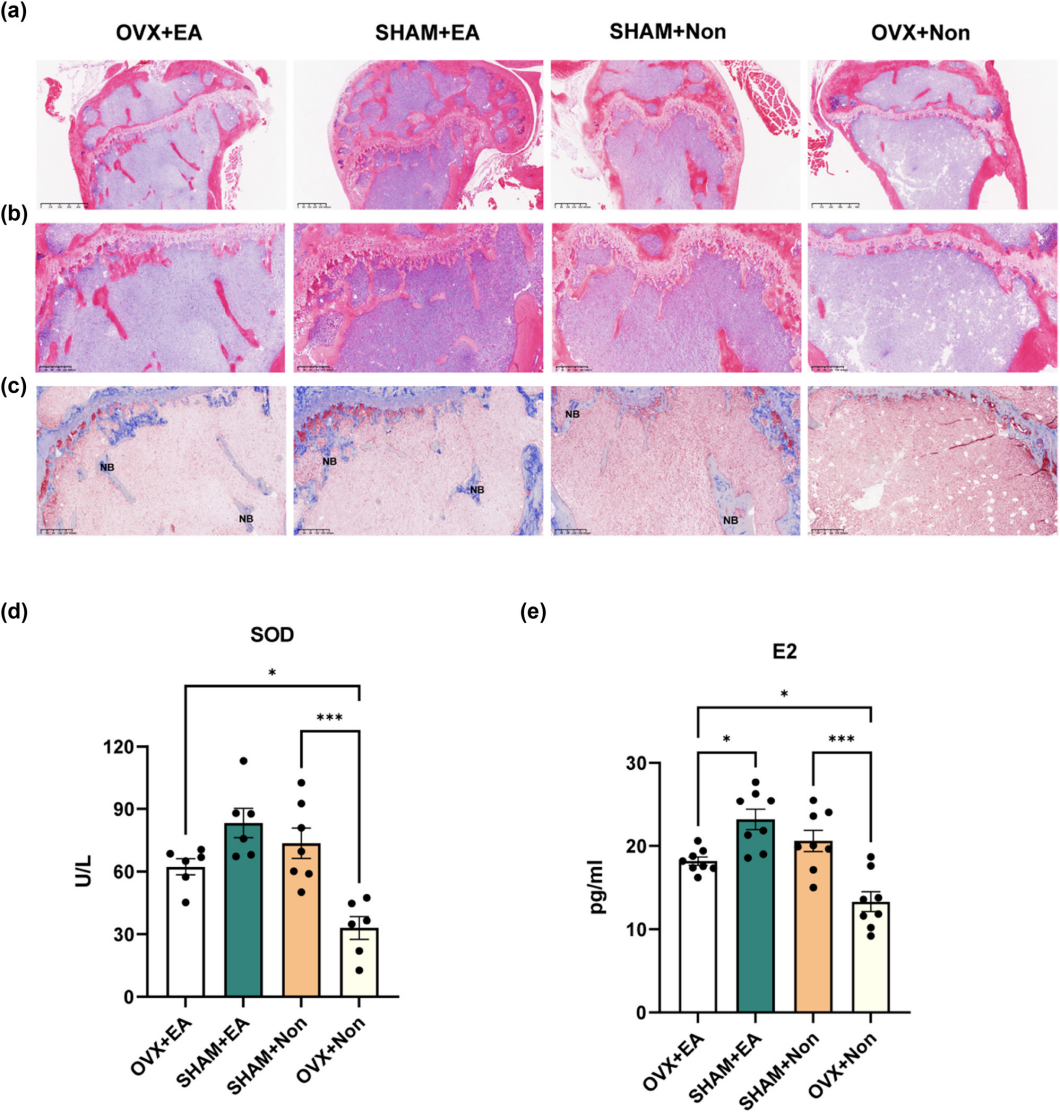
Bone histopathology and detection of serum indexes in mice. The Hematoxylin-Eosin staining of the four groups under 4× scope (a). The Hematoxylin-Eosin staining of the four groups under 10× scope (b). The Masson staining of the four groups under 10× scope; blue shows newly formed bone, and red shows mature bone tissue; NB represents new bone trabeculae (c). Four groups of mice, each group (*n* ≥ 6) were included in the test. The *X*-axis is the group, and the *Y*-axis is the concentration in the serum (d and e). Data are presented as mean ± SEM; *, *p* < 0.05; ***, *p* < 0.001.

A histological examination using Hematoxylin-Eosin staining and Masson staining revealed distinct changes in bone quality in the OVX + non group. Specifically, a reduction in the trabecular number, a disrupted and discontinuous trabecular arrangement, fractured trabeculae, and significant expansion of the bone marrow cavity were observed. Additionally, an increase in fat cell density and decreased osteoblasts were evident. However, these pathological features were ameliorated in ovariectomized mice receiving the EA intervention (Figure 3a–c).

### 3.2 EA increased the concentration of SOD and estradiol in serum

Oxidative stress is a major contributor to bone loss, and its impact on bone health has been extensively studied. In this study, we examined the expression of SOD in the serum of model mice and found that EA significantly increased SOD levels, whereas SOD levels decreased when mice underwent ovariectomy. We further compared the OVX + non group with the SHAM + non group and found that oxidative stress increased after ovariectomy (Figure 3d). Estradiol is critical for osteoblast formation and differentiation, and our study revealed that the serum estradiol levels in ovariectomized mice were significantly increased in the EA groups compared to the non groups (Figure 3e). Our results suggest that the loss of estradiol reduces the defense against oxidative stress in bone, while EA treatment inhibits bone injury by increasing serum estradiol levels. Therefore, our findings support the potential of EA as a therapeutic approach to prevent bone loss induced by oxidative stress in ovariectomized mice.

### 3.3 Estradiol inhibits bone damage by activating the PI3K–Akt signaling pathway

**Figure 4:**
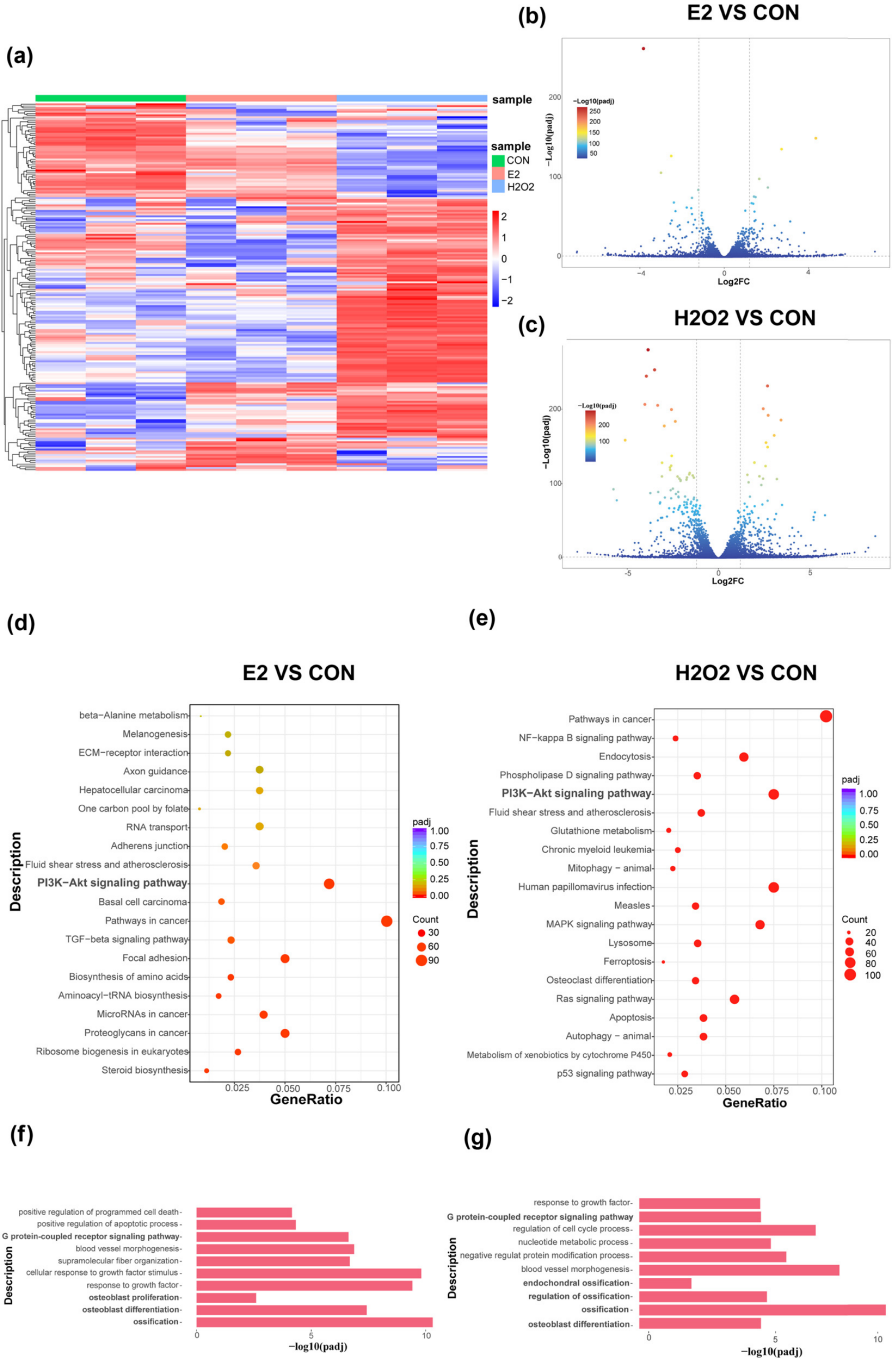
Transcriptome results of MC3T3-E1 treated with estradiol and H_2_O_2_ respectively. Gene expression heatmap; mRNA levels changed significantly under two treatments. Color-coded microarray hybridization signals (blue to red = low-to-high signals) of the H_2_O_2_ group, the CON group, and the estradiol group (a). The volcanic plot, deferentially expressed genes in the two comparisons (estradiol group versus CON group, H_2_O_2_ group versus CON group) are clustered as dots in the figure. Two vertical lines indicate gene expression fold change >1 and <−1, respectively, and the horizontal line indicates the adjusted *p* value of 0.05. *p* values were calculated by a two-sided Wilcoxon rank-sum test. The color of the dot represents the *p* adjust levels (b and c). As shown in the figure, the abscissa is the ratio of the number of differentially expressed genes annotated to the total number of differentially expressed genes, and the ordinate is the KEGG pathway. The size of the dots represents the number of genes annotated to the KEGG pathway, and the color from red to blue represents the significance of enrichment (d) and (e). The PI3K–Akt pathway in (e) and (f) pictures was marked in blod font. Differential genes were enriched in the GO database. As shown in the figure, ten GO terms with more enriched genes were drawn from the BP ontologies. The horizontal coordinate is the description of the enriched gene, and the vertical coordinate is −1og10(*p*adj) (f and g). Descriptions related to bone are highlighted.

MC3T3-E1 cells were subjected to treatment with H_2_O_2_ and estradiol, followed by transcriptome analysis using cluster analysis, differential gene volcano, KEGG enrichment, and Gene Ontology (GO) enrichment. Cluster analysis of the differentially expressed gene sets revealed that genes with similar expression patterns were clustered together, suggesting potential common functions or involvement in common metabolic and signaling pathways. The colors in the heatmap can only be compared horizontally (the expression of the same gene in different samples). We identified differential expression of genes involved in osteoblast generation and differentiation (Figure 4a). The volcano plot depicted the differential expression of genes involved in osteoblast generation and differentiation. More genes were differentially expressed with H_2_O_2_ treatment compared to estradiol treatment (Figure 4b and c). Then, we performed qPCR on several genes associated with osteoblast differentiation. From the results of the KEGG enrichment, the 20 most significant KEGG pathways were selected for generating scatter plots. Through KEGG enrichment, we found that the PI3k–Akt signaling pathway changed significantly and was downregulated in the H_2_O_2_ group and upregulated in the estradiol group (Figure 4d and e). GO is an internationally standardized classification system of gene functions used to comprehensively describe the properties of genes and gene products in living organisms. It categorizes genes according to molecular function, cellular component, and biological process (BP). The results of the GO analysis showed that the BPs of the genes in the two groups were mostly focused on osteoblast proliferation, differentiation, ossification, cell cycle, apoptosis, and the G protein-coupled receptor (GPCR) signaling pathway (Figure 4f and g). The results suggested that the PI3K–Akt pathway may play a crucial role in mediating the effects of H_2_O_2_ and estradiol on osteoblast differentiation and proliferation, as supported by the significant number of enriched genes associated with this pathway and their significant *p*-value.

### 3.4 Estradiol upregulates the expression of genes related to bone formation

**Figure 5:**
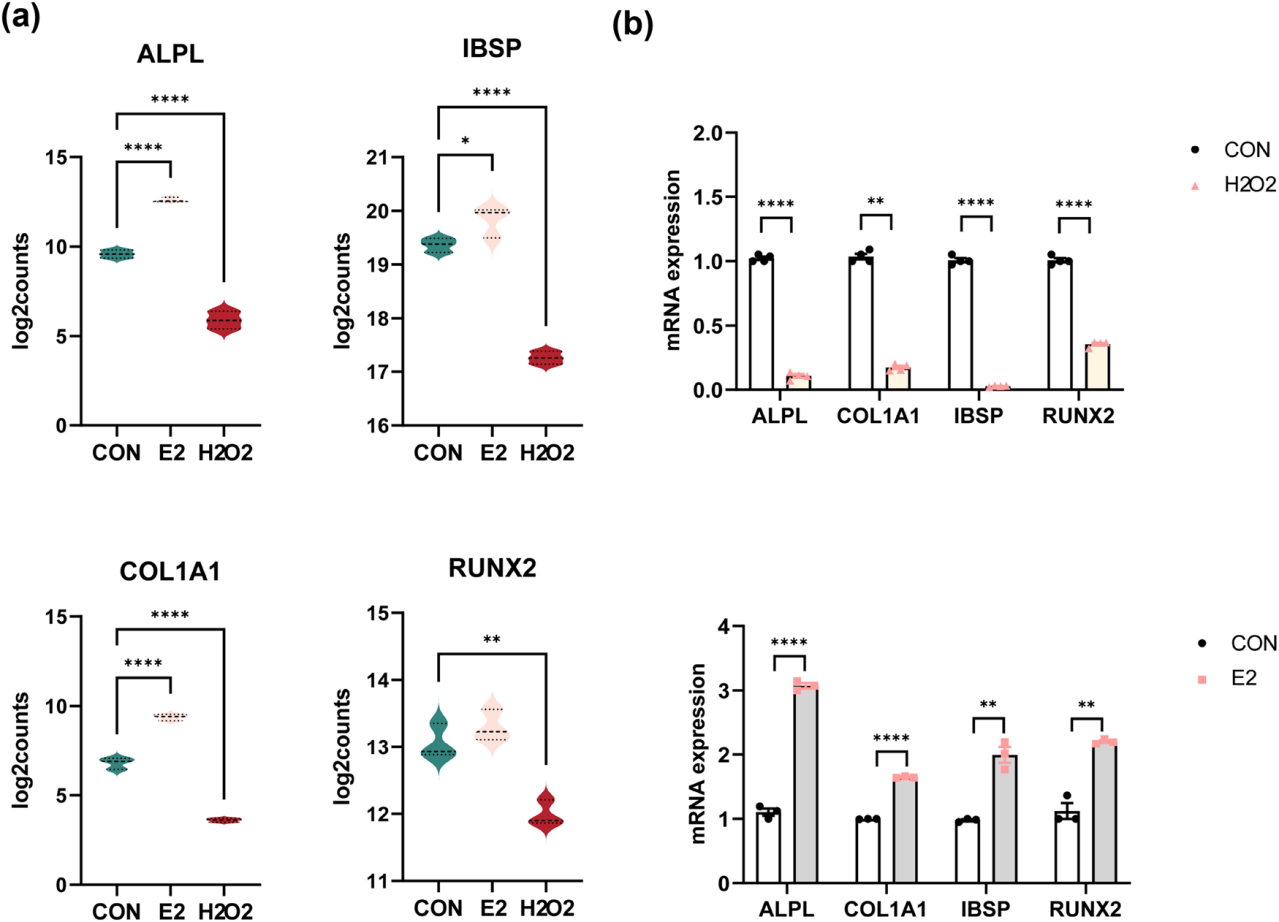
Expression of genes associated with bone formation. In transcriptome sequencing, the number of counts alignment on the four genes Alpl, Ibsp, Col1a1, and Runx2 indicated the expression level (a). H_2_O_2_ decreases the differentiation of osteoblast-related genes, and osteoblasts were pretreated with H_2_O_2_ (100 µm) for 12 h. Estradiol increases the differentiation of osteoblast-related genes, and osteoblasts were pretreated with Estradiol (10^−6^ mol/L) for 12 h. The mRNA expression value is normalized to GAPDH expression (b). The data are presented as the mean ± SEM, *, *p* < 0.05; **, *p* < 0.01; ****, *p* < 0.0001.

Alkaline phosphatase (*Alpl*), bone sialoprotein (*Ibsp*), type Ⅰ collagen (*Col1a1*), and runt-related transcription factor 2 (*Runx2*) are well-known genes that play important roles in osteoblastic differentiation [41,42]. *Alpl* has been widely used as a diagnostic index to evaluate the bone formation capacity in osteoporosis due to its well-established use as an osteoblastic marker. Ibsp serves as an intermediate-stage marker, while *Col1a1* is an early marker of osteoblast differentiation as it is primarily produced by osteoblasts during bone formation. Runx2 is involved in inhibiting adipogenic differentiation and directing mesenchymal stromal cells toward an osteogenic lineage [41,42]. Our transcriptome analysis revealed that the expression of these four genes was differentially regulated (Figure 5a). Subsequent qPCR experiments unequivocally substantiated that these genes were upregulated following estradiol administration but were downregulated after H_2_O_2_ treatment (Figure 5b). The consistency between the results obtained from the transcriptome analysis and the qPCR experiments supports the differential expression findings of these four genes in response to H_2_O_2_ and estradiol treatments during osteoblastic differentiation. This evidence showed that estradiol impeded bone loss and fostered bone formation.

### 3.5 EA treats osteoporosis by activating the PI3K–Akt pathway

**Figure 6:**
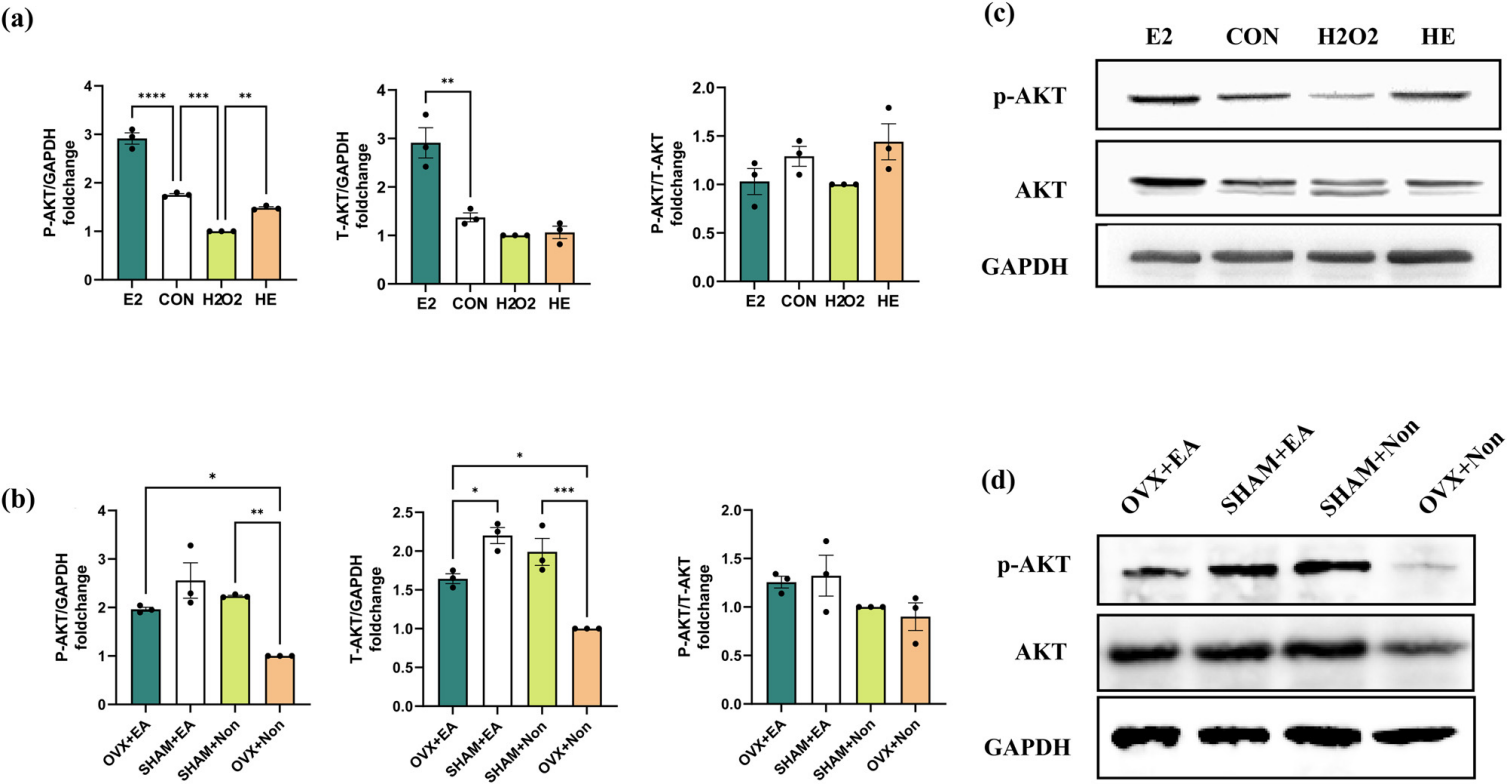
Changes in proteins of the PI3K-Akt pathway under different treatments. Cells were pretreated with H_2_O_2_, E2, and H_2_O_2_ + E2 for 12 h. AKT and P-AKT levels were measured by Western blot. E2, estradiol; CON, control; HE, H_2_O_2_ + E2. Quantitative analysis of the Western blot (*n* = 3); the relative value is normalized to GAPDH expression. The data are presented as the mean ± SEM. *, *p* < 0.05; **, *p* < 0.01; ***, *p* < 0.001; ****, *p* < 0.0001 (a, b). Western blot analysis for the expression of AKT and P-AKT at the cellular level (c); Western blot analysis for the expression of AKT and P-AKT at the tissue level (d).

Transcriptome analysis revealed that the PI3K–Akt pathway is involved in bone resorption induced by oxidative stress. To investigate whether estradiol could inhibit bone mass loss caused by oxidative stress, we assessed the effect of estradiol on p-AKT expression at the cellular level. Estradiol increased p-AKT expression, while H_2_O_2_ decreased it. Estradiol treatment of the H_2_O_2_ treatment group partially restored the decreased p-AKT expression. The p-AKT level was significantly changed in the estradiol-treated group, H_2_O_2_-treated group, and H_2_O_2_+E2 (HE)-treated group, and total AKT was significantly changed in the estradiol-treated group. However, the ratio of phosphorylated AKT to total AKT was not significant in any of the groups (Figure 6a and c). At the tissue level, we explored the effect of EA on bone quality and the PI3K–Akt signaling pathway. Compared to the SHAM + non group, the OVX + non group exhibited a reduction in p-AKT expression post-ovariectomy, which was subsequently increased following EA intervention, as observed in the OVX + EA group. A similar trend was observed in the level of total AKT protein; however, there was no significant difference in the ratio of phosphorylated AKT to total AKT (Figure 6b and d). These findings indicate that EA confers significant protection against oxidative stress-induced injury in osteoblast cells, potentially by activating the PI3K–Akt pathway.

## 4 Discussion

With the rapid progression of global population aging, the incidence of osteoporosis is steadily rising, particularly among postmenopausal women. Given the significant morbidity and mortality rates associated with osteoporosis and its substantial social and economic burden, osteoporosis has become a significant public health concern [43]. Existing treatment modalities are frequently inadequate and are hindered by potential side effects and suboptimal patient adherence. Thus, this study sought to elucidate the therapeutic effects of EA, a TCM intervention, as a complementary therapy for osteoporosis. EA is cost-effective, has minimal side effects, and has shown promise in the management of osteoporosis.

In this study, we demonstrated that EA ameliorated osteoporosis in ovariectomized mice, as evidenced by significant improvements in bone histomorphometry and tissue histology. Furthermore, we showed that EA increased systemic estrogen levels in ovariectomized mice. The beneficial effects of EA on osteoporosis in ovariectomized models have been previously reported, in which BMD, bone strength, bone structure, and markers of bone formation and resorption all improved [29]. Consistent with prior research, our findings underscore the therapeutic potential of EA in alleviating osteoporosis. Despite variations in the types of acupuncture and the selection of specific acupoints compared to previous studies, our results highlight the necessity for further exploration into the mechanisms underlying the effects of EA.

Acupuncture, originating in China and practiced for over 2,500 years, has recently garnered scientific interest for its potential regulation of the “neuro-endocrine-immune network,” a concept introduced by Professor Besedovsky in the 20th century. Acupuncture can locally activate cell functions and neuroreceptors and also regulate the release of biomolecules, such as hormones, neurotransmitters, and neuromodulators into the microenvironment, thus achieving comprehensive regulation through their interactions [44]. Previous studies demonstrated that acupuncture can modulate the hypothalamic–pituitary–gonadal (adrenal) axis. In ovariectomized rats, acupuncture increased serum levels of estradiol, parathyroid hormone, bone gla protein, insulin-like growth factor-1, and calcitonin, thereby enhancing calcium metabolism and bone homeostasis [45]. These findings suggest that hormonal regulation is a direct effect of EA stimulation, consistent with our results.

According to TCM theory, osteoporosis is primarily caused by Qi deficiency and blood stasis, which are often associated with dysfunction in the liver, kidney, and spleen [46]. Numerous studies have explored acupuncture treatment for postmenopausal osteoporosis from the perspective of liver and kidney deficiency, focusing on specific acupoints such as BL23 (*Shenshu*), ST36 (*Zusanli*), BL11 (*Dazhu*), CV4 (*Guanyuan*), GV4 (*Mingmen*), SP6 (*Sanyinjiao*), KI3 (*Taixi*), BL18 (*Ganshu*), and GB39, consistent with the principles of nourishing the kidney and spleen [45]. A previous study reported that EA treatment at ST36 and SP6 acupoints increased serum estradiol levels, BMD, and bone strength in ovariectomized rats [47]. Additionally, EA treatment at BL20 (*Pishu*), BL23, and ST36 acupoints in ovariectomized New Zealand rabbits prevented bone loss by upregulating estradiol and downregulating osteoprotegerin (OPG) ligands [48]. Based on the “*Shaoyang* main bone” theory recorded in the *Neijing*, some researchers suggest that acupuncture at the *Shaoyang* meridian points of the foot can balance osteogenesis and osteoclastic resorption, thus improving osteoporosis [36]. Consequently, in our study, we selected the GB30, GB34, and GB39 acupoints, all key points of the *Shaoyang* gallbladder meridian, to further investigate the efficacy of EA applied to these acupoints for treating osteoporosis, and to discern the underlying molecular mechanisms.

The mechanisms underlying the efficacy of acupuncture in treating osteoporosis have been partially elucidated. First, acupuncture appears to mitigate bone loss by modulating the adrenal axis. Previous research demonstrated that acupuncture at ST36, BL23, and BL11 acupoints regulated serum levels of estradiol, corticotropin-releasing hormone, adrenocorticotropin, and corticosterone, thereby improving osteoporosis [49]. Second, acupuncture has been shown to reduce pro-inflammatory factors, inhibiting those involved in osteoclastogenesis and enhancing bone metabolism. Studies have indicated that acupuncture at the ST36 acupoint reduced serum IL-1β expression in ovariectomized rats; generally, acupuncture can mitigate bone destruction and slow the progression of osteoporosis by downregulating inflammatory factors such as tumor necrosis factor-α and interleukin-6 [45,50]. Third, acupuncture activates various signaling pathways that promote bone formation and increase bone density. For example, acupuncture has been shown to regulate the RANKL–OPG pathway to inhibit bone resorption, activate the Wnt/β-catenin pathway to promote bone formation and strengthen bone, and modulate the TGF-β signaling pathway to enhance osteoblast proliferation [29,45,51]. Notably, the role of the PI3K–Akt pathway in acupuncture-induced effects on ovariectomized mice has remained unexplored, and our study addresses this gap in the literature.

The PI3K–Akt pathway is well-documented as promoting osteoblast proliferation and inhibiting bone resorption [52]. Inhibition of p-AKT exacerbates bone mass loss in osteoarthritis models, while upregulation of AKT and particularly of p-AKT by estradiol is known to induce chondrocyte proliferation [53]. This pathway is also involved in regulating the proliferation, differentiation, and apoptosis of osteoclasts and osteoblasts. Activation of the PI3K–Akt signaling pathway in ovariectomized mouse models was shown to improve mitochondrial function, alleviate osteoblast pyroptosis, promote bone formation, and ameliorate osteoporosis [54]. Additionally, activation of the PI3K–Akt pathway promoted the proliferation and differentiation of bone marrow mesenchymal stem cell precursors in ovariectomized rats [55]. The importance of Akt in bone formation is highlighted by *Akt* knockout (Akt-KO) mice, which exhibit osteopenia, reduced body size, and shorter bone length [56]. Our study corroborated these findings and demonstrated that estradiol inhibited H_2_O_2_-induced bone loss via the PI3K–Akt pathway in ovariectomized mice. This underscores the role of the PI3K–Akt pathway in counteracting oxidative stress-induced bone loss, which is particularly significant given the detrimental impact of reactive oxygen species on bone health. Although previous studies have primarily used rats as research models, we opted for mice due to their ease of handling and the use of osteogenic cell lines also derived from mouse skulls. Additionally, mice share closer genetic and physiological similarities to humans, potentially enhancing the clinical translatability of our findings.

**Figure 7:**
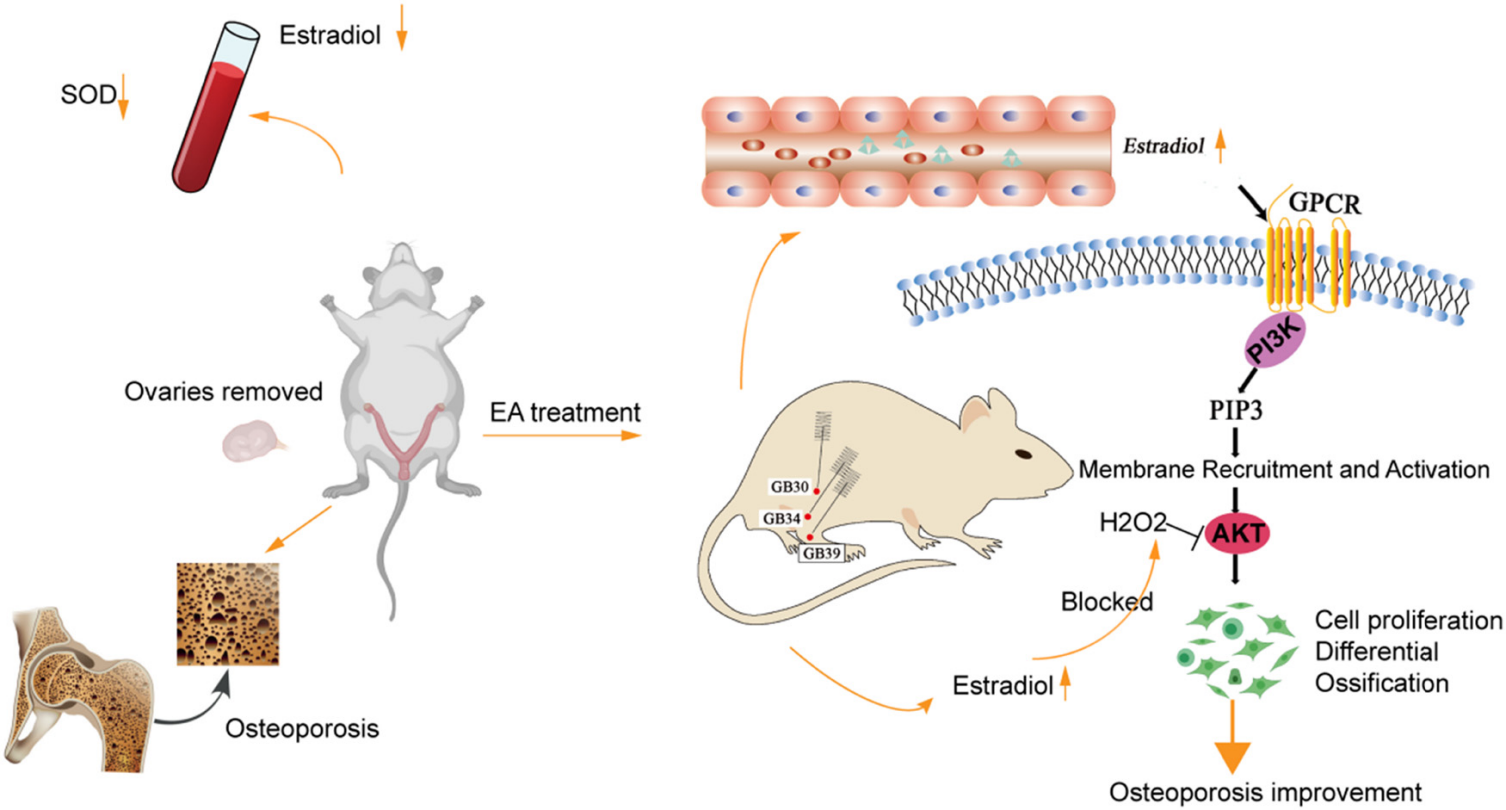
Schematic diagram demonstrating how electroacupuncture improves osteoporosis.

The role of GPCR, also known as G protein-coupled estrogen receptor, in the proliferation of human fetal osteoblast cell lines induced by estradiol is well-documented [57]. Estrogen-induced GPCR is crucial in the osteogenic axis, as it activates the ERK1/2 and PI3K–Akt signaling pathways to promote mitophagy in osteoblasts and reduce osteoblast apoptosis, thereby reversing osteoporosis [54,58,59]. Our GO enrichment analysis further corroborated that the GPCR signaling pathway is implicated in these biological processes (Figure 4f and g). In our study, ovariectomized mice developed osteoporosis, characterized in part by decreased serum SOD and estradiol levels. Subsequent EA stimulation at acupoints GB30, GB34, and GB39 increased serum estradiol levels in these mice, effectively mitigating bone damage induced by oxidative stress. Estradiol exerts its effects by binding to GPCR on cell membranes and activating PI3K, which subsequently activates Akt. This activation promotes cell differentiation, proliferation, and ossification, ultimately attenuating osteoporosis (Figure 7). Our findings contribute to the growing body of evidence supporting the beneficial effects on bone health of applying EA at GB acupoints and provide insights into the underlying mechanisms involved.

Despite these encouraging findings, there are still some limitations that need to be addressed. Experiments on mice may affect the applicability of the results in clinical settings. We look forward to conducting future studies on larger animal models, such as rabbits, dogs, pigs, or primates, to provide a molecular basis for future clinical applications. Moreover, investigating how different acupoints might interact with various signaling pathways involved in bone metabolism could provide a more comprehensive understanding of the mechanisms by which EA improves osteoporosis. Furthermore, longitudinal studies to observe the long-term effects of EA on bone health and its potential preventative benefits would be highly valuable.

## 5 Conclusion

This study elucidated the role of the PI3K–Akt signaling pathway in the therapeutic efficacy of EA at specific GB acupoints for treating osteoporosis. Specifically, the PI3K–Akt signaling pathway was observed to be attenuated in osteoporotic mice, and EA stimulation at specific GB acupoints markedly increased the expression of p-AKT and serum estradiol in these mice. These findings suggest that EA exerts its molecular effects in the treatment of osteoporosis by enhancing osteoblast differentiation, proliferation, and ossification by upregulating serum estradiol and activating p-AKT.
